# Hemolysis during and after 21 days of head‐down‐tilt bed rest

**DOI:** 10.14814/phy2.13469

**Published:** 2017-12-21

**Authors:** Guy Trudel, Hans K. Uhthoff, Odette Laneuville

**Affiliations:** ^1^ The Ottawa Hospital Rehabilitation Centre Ottawa Ontario Canada; ^2^ University of Ottawa Faculty of Medicine Department of Medicine Ottawa Ontario Canada; ^3^ Ottawa Hospital Research Institute Clinical Epidemiology Program Ottawa Ontario Canada; ^4^ Faculty of Medicine University of Ottawa Ottawa Ontario Canada; ^5^ Department of Biology Faculty of Science University of Ottawa Ottawa Ontario Canada

**Keywords:** Carbon monoxide, head‐down‐tilt bed rest, hemolysis, urobilinogen

## Abstract

Hemoconcentration is observed in bed rest studies, descent from altitude, and exposure to microgravity. Hemoconcentration triggers erythrocyte losses to subsequently normalize erythrocyte concentration. The mechanisms of erythrocyte loss may involve enhanced hemolysis, but has never been measured directly in bed rest studies. Steady‐state hemolysis was evaluated by measuring two heme degradation products, endogenous carbon monoxide concentration [CO] and urobilinogen in feces, in 10 healthy men, before, during, and after two campaigns of 21 days of 6° head‐down‐tilt (HDT) bed rest. The subjects were hemoconcentrated at 10 and 21 days of bed rest: mean concentrations of hemoglobin (15.0 ± 0.2 g/L and 14.6 ± 0.1 g/L, respectively) and erythrocytes (5.18 ± 0.06E6/*μ*L and 5.02 ± 0.06E6/*μ*L, respectively) were increased compared to baseline (all *P*s < 0.05). In contrast, mean hemoglobin mass (743 ± 19 g) and number of erythrocytes (2.56 ± 0.07E13) were decreased at 21 days of bed rest (both *P*s < 0.05). Indicators of hemolysis mean [CO] (1660 ± 49 ppb and 1624 ± 48 ppb, respectively) and fecal urobilinogen concentration (180 ± 23 mg/day and 199 ± 22 mg/day, respectively) were unchanged at 10 and 21 days of bed rest compared to baseline (both *P*s > 0.05). A significant decrease in [CO] (−505 ppb) was measured at day 28 after bed rest. HDT bed rest caused hemoconcentration in parallel with lower hemoglobin mass. Circulating indicators of hemolysis remained unchanged throughout bed rest supporting that enhanced hemolysis did not contribute significantly to erythrocyte loss during the hemoconcentration of bed rest. At day 28 after bed rest, decreased hemolysis accompanied the recovery of erythrocytes, a novel finding.

## Introduction

Head‐down‐tilt (HDT) bed rest has been used to mimic some adaptive and recovery effects of microgravity (Hargens et al. 2013). HDT bed rest induces a shift in body fluids which decreases intravascular plasma volume and concentrates erythrocytes (Hargens et al. 2013; Bilancio et al. 2014; Otto et al. 2017). The adaptive response to this hemoconcentration involves the loss of erythrocytes that can reach clinical anemia levels (Trudel et al. 2009). The mechanisms of bed rest‐induced loss of erythrocytes have not been previously investigated and may involve enhanced hemolysis, over and above the removal of senescent erythrocytes.

Hemoconcentration and erythrocyte losses have also been reported in microgravity (Rizzo et al. 2012). Historically space anemia has been explained by a number of possible mechanisms: decreased erythropoietin (EPO) production, erythropoietic suppression, sequestration, blood loss, and enhanced hemolysis (Tavassoli 1982). One group suggested rapid destruction of young erythrocytes (neocytolysis) to resolve hemoconcentration in space, descent from altitude, withdrawal from EPO administration, and renal disease (Alfrey et al. 1996, 1997; Rice et al. 1999, 2001; Chang et al. 2009; Pottgiesser et al. 2012). Franco (2009) pointed out that the evidence for neocytolysis was indirect. We therefore set out to measure direct indicators of hemolysis during the hemoconcentration of bed rest and up to 28 days after bed rest.

Metabolism of hemoglobin by the heme oxidase enzyme can be quantified directly via the stoichiometric 1:1 cleavage of one molecule of heme into one molecule of biliverdin plus one molecule of carbon monoxide (CO) (Heinemann et al. 2014). CO is exchanged in gaseous form in the lung alveoli down a concentration gradient. Endogenous CO elimination has been measured by end‐tidal or alveolar CO concentration ([CO]) and reflected hemolysis rates in health and disease (Tavassoli 1982; Franco 2009; Coburn 2012; Levitt and Levitt 2015). Biliverdin, in turn, is reduced into bilirubin, conjugated in the liver, and degraded by host bacteria into urobilinogen, minimally reabsorbed by the enterohepatic circulation, and mainly excreted in the feces and less so in the urine (Beris and Picard 2015). Increased urobilinogen production also reflected hemolysis rates (Berlin 1975; Kotal and Fevery 1991).

Our objective was to measure indicators of hemolysis (haptoglobin, endogenous CO elimination, bilirubin, and urobilinogen), of hemoconcentration (blood volume, erythrocyte concentration), and of erythrocyte production (reticulocytes) (Doig 2015) in 10 healthy men before, during, and up to 28 days after they were subjected twice to 6° HDT 21‐day bed rest. Our hypotheses were that: (1) bed rest causes hemoconcentration without a significant increase in hemolysis and (2) erythrocyte loss regenerates 28 days after bed rest through enhanced stimulation.

## Materials and Methods

### Subjects

**Figure 1:**
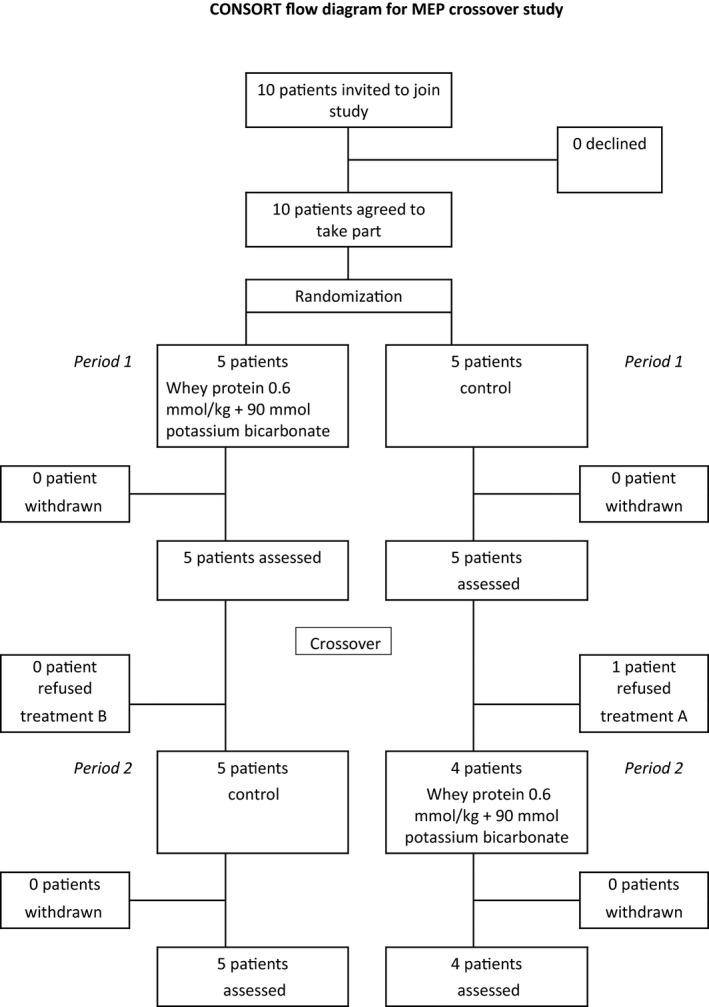
CONSORT diagram of the study.

The study protocol was approved by the ethics commission of the Aerztekammer Nordrhein (Düsseldorf, Germany) and registered in ClinicalTrials.gov (#NCT01655979). Under the leadership of the German Space Agency (DLR), a bed rest study was prepared to investigate the effect of a combined whey protein (0.6 g/kg body weight/day) and potassium bicarbonate (90 mmol/day) supplementation as a potential countermeasure to multiple physiological and metabolic alterations on the human body resulting from simulated microgravity. International research teams were selected to measure physiological and metabolic alterations. Our team investigated hemoconcentration and hemolysis. A detailed protocol for subject recruitment, exclusion criteria, and nutritional intervention appeared in a separate publication (Buehlmeier et al. 2014). Ten healthy men aged between 20 and 45 years gave written informed consent to undergo two campaigns of 21 days of 6° HDT bed rest. This cross‐over design tested the effect of a 1:1 allocation to receive the whey protein‐ and potassium bicarbonate‐supplemented diet or a control diet, separated by a 4‐month wash‐out period (Fig. 1). Participants were involved in multiple experiments besides the current protocol, including muscle biopsies.

### Methods

Both campaigns took place at the German Aerospace Center, Köln, Germany. The first campaign was conducted in September and October 2011; the second campaign was conducted in February and March 2012. Study design included 8 days of environmental and dietary adaptation for baseline data collection named BDC8 to BDC1, 21 days of HDT bed rest named HDT1 to HDT21, 5 days of recovery named R0 to R5 consisting of reambulation before discharge, and a follow‐up visit at R28. During BDC, participants were discouraged from lying in bed during the day.

### Blood Sampling

In each campaign, 596 mL of blood was drawn per subject over the 56 days duration of each campaign. This included 441 mL drawn between BDC5 and HDT21, or 17.6 mL/day at 14.5 g/dL of Hb; venipuncture corresponded to an estimated loss of 2.5 g Hb/day. All blood was drawn in the bedridden position. Assays were performed at MLM Medical (Moenchengladbach, Germany). Hematocrit, hemoglobin, and erythrocyte concentrations were measured using a Counter ABXpentra60Cplus. Reticulocytes were counted manually. Haptoglobin was measured by nephelometric assay (Siemens, Marburg, Germany). Total and direct/conjugated bilirubins were measured using photometric assays, ferritin by electrochemiluminescence assay, C‐reactive protein by immunoturbidimetric assay (Roche, Mannheim, Germany), and EPO by chemiluminescent immunoassay (Beckman, Krefeld, Germany).

### Blood volumes

Total hemoglobin mass (tHb), blood volume, plasma volume, and erythrocyte volume were estimated using CO‐rebreathing at BDC5 and HDT21 according to the protocol by Schmidt and Prommer (2005). In summary, an individualized bolus of CO was rebreathed for 7 min and the kinetics of HbCO formation enabled the calculation of tHb using the following formula: tHb = (K × CO × 0.986) × 100/ΔCOHb × 1.39, where CO is the adsorbed CO, 0.986 the myoglobin correction, ΔCOHb is the difference in carboxyhemoglobin, 1.39 the oxygen binding capacity of Hb, and K the environment correction factor. tHb was used to calculate the number of mature erythrocytes and reticulocytes per subject. Erythrocyte volume = tHb/mean corpuscular hemoglobin concentration × 100. Blood volume = Erythrocyte volume × (100/Ht × 0.91), where Ht is the hematocrit and 0.91 the correction for Fahraeus effect (Schmidt and Prommer 2005). Finally, plasma volume = blood volume − erythrocyte volume.

### Number of mature and immature erythrocytes

Erythrocytes comprised immature erythrocytes (reticulocytes) and mature erythrocytes. In order to appreciate if one population of erythrocytes was preferentially affected, we estimated the number of immature and mature erythrocytes per subject using the following calculations: (1) number of erythrocytes = tHb/mean corpuscular hemoglobin, (2) number of reticulocytes = %reticulocytes × number of erythrocyte, and (3) number of mature erythrocytes = number of erythrocytes − number of reticulocytes (Schmidt and Prommer 2005).

### Alveolar and ambient air samples

Alveolar and ambient air samples at BDC1, HDT5 (campaign 2 only), HDT10, HDT21, and R1 were collected at the bedside upon awakening at 0630 h. This ensured air sampling was always carried out before a CO‐rebreathing blood volume measurement. HDT5 time point was added at campaign 2 to rule out interval hemolysis between BDC2 and HDT10. Morning sampling allowed approaching a steady‐state between endogenous CO elimination, blood COHb, CO diffusion capacity, alveolar PCO, and alveolar ventilation (Coburn 2012). An airtight 50‐mL syringe was filled with ambient air at the bedside. The measure of ambient air [CO] will account for environmental contaminations of alveolar air (Franco 2012). Alveolar air was collected after a 20‐sec breath holding at the peak of a normal inspiration. On expiration, the first 400 mL was diverted into a discard bag and the remainder was directed through a one‐way valve into a 750‐mL collection bag (QuinTron, Milwaukee, WI). The alveolar air collection was repeated.

Alveolar and ambient air samples were analyzed the same morning using a gas chromatograph (GC) with a reduction gas detector (Ametek, Newark, NJ). GC was calibrated with 1500 ppb [CO] and used 99.9999% pure nitrogen as carrier gas. Three readings of ambient air and six readings of alveolar air (three readings per bag) at each time point were averaged.

Subjects returned to the bed rest facility at R28. They had collected alveolar and ambient air samples at home upon awakening. Alveolar and ambient air samples were repeated upon arrival at the facility. Both R28 (home) and R28repeat (bed rest facility) data are presented. The endogenous [CO] is the difference between alveolar air [CO] and ambient air [CO]. Endogenous [CO] corresponds to the elimination rate of endogenously produced CO.

### Preparation of fecal suspensions and urine samples

Feces were collected over 24 h, weighed, immediately frozen at −20°C, and protected from light. Five 3‐day stool collections were performed (BDC3‐2‐1, HDT5‐6‐7, HDT12‐13‐14, HDT19‐20‐21, and R2‐3‐4). Frozen stools were thawed in a cool room under dim lighting and homogenized, and a 350 mg sample was mixed with 15 mL of a 0.16 mmol/L NaCl solution. One mL of the fecal suspension was extracted, protected from light, and frozen at −20°C. Twenty‐four‐hour urine samples were collected on the same days, put on ice, and protected from light. A 2.4‐mL aliquot of urine was stored at −20°C, protected from light.

### Measure of urobilinogen

Urobilinogen concentrations in fecal suspensions and urine samples were measured by spectrophotometry. The oxidation products of urobilinogen–zinc complexes were extracted from 0.4 mL of fecal suspensions and from 2.4 mL of urine by adding to each sample: 2.4 mL of a zinc acetate solution (54 mmol/L in dimethylsulfoxide), 0.2 mL of iodine (25 mmol/L in a 120 mmol/L potassium iodine solution), and 0.1 mL of cysteine (82 mmol/L in water) (Kotal and Fevery 1991). Samples were mixed vigorously, centrifuged at 5000 g for 3 min, and the first supernatant collected. Each pellet underwent a repeat extraction. A calibration curve ranged from 1 to 60 *μ*mol/L of urobilinogen (Lee BioSolutions, St. Louis, MO) dissolved in dimethylsulfoxide plus zinc acetate, iodine, and cysteine solutions added at same final concentrations as the samples. The concentration of urobilinogen was calculated by measuring the optical density at 508 nm of the two supernatants generated from each sample, normalized to the 24 h weight of feces and to the 24 h volume of urine.

### Calculation of the expected rise in endogenous [CO] if tHb loss were hemolysed

At BDC5, the turnover rate of hemoglobin was estimated at 838 g × 1/120 days (assuming erythrocyte life span of 120 days) = 7 g/day or 108.5 *μ*mol Hb/day (molar weight Hb: 64,500 g/mol). One molecule of heme catalyzed to biliverdin produces 1 molecule of CO. Four molecules of heme form 1 molecule of Hb; this 1:4 stoichiometry predicts that catabolism of 108.5 *μ*molHb/day produces × 4 = 434 *μ*mol CO/day. Of endogenous CO, 85% is assumed to originate from Hb degradation. At BDC5, endogenous CO elimination was calculated at 434 *μ*mol/day/0.85 = 510 *μ*mol CO/day.

At BDC2, the elimination of 510 *μ*mol CO/day at 1685 ppb[CO] required an alveolar ventilation of 510 *μ*mol CO/day × 22.4 L/mole of air/1685 ppb[CO] = 6780L/day, where 22.4 L is the volume of 1 mole of air at standard temperature and pressure dry and 1685 ppb[CO] is the endogenous [CO] measured at BDC.

During bed rest, tHb loss of 3.8 g Hb/day–2.5 g Hb/day from venipuncture − 0.4 g Hb/day from reduced reticulocyte production = 0.9 g Hb/day could have hemolysed. According to the above calculations, hemolysis of 0.9 g Hb/day would increase CO elimination by approximately 56 *μ*mol CO/day and endogenous [CO] by 185 ppb compared to BDC2.

These calculations accounted for venipuncture and decreases in reticulocyte numbers, and assumed stable alveolar ventilation and turnover of nonheme molecules.

### Statistical methods

Mean ± 1 SEM of all subjects’ data are displayed. A repeated measure ANOVA explored the main effects of nutrition intervention (diet/control), campaign (1 and 2), and duration of bed rest. After validation for sphericity based on Mauchly's test, significant main effects and interactions were examined using post hoc pairwise multiple comparisons with a Fisher's least square difference correction for multiple comparisons. Endogenous [CO] at HDT5 of the second campaign included nine patients who were compared with their baseline values using a paired *t*‐test. Comparisons reaching a *P*‐level of 0.05 using SPSS 24 (IBM, Armonk, NY) were considered statistically significant.

## Results

Subjects’ mean age was 32 years and mean BMI 23.2 kg/m^2^. One subject discontinued the study after the first campaign for medical reasons; the data collected during the first campaign were analyzed. Repeated measures ANOVA showed no effect of nutrition intervention (diet/control) on any reported hematological outcome. There was also no effect of campaign (1 and 2) on the main outcomes measured. However, there was a main effect of duration of bed rest which was further examined as a secondary analysis. Testing the effect of diet was the main purpose of the crossover design. Since the dietary intervention proved to have no effect, the two campaigns were analyzed and presented as repeat experiments including all outcome measures on all available subjects at all available time points.

### Hemoglobin and erythrocyte concentrations

**Figure 2:**
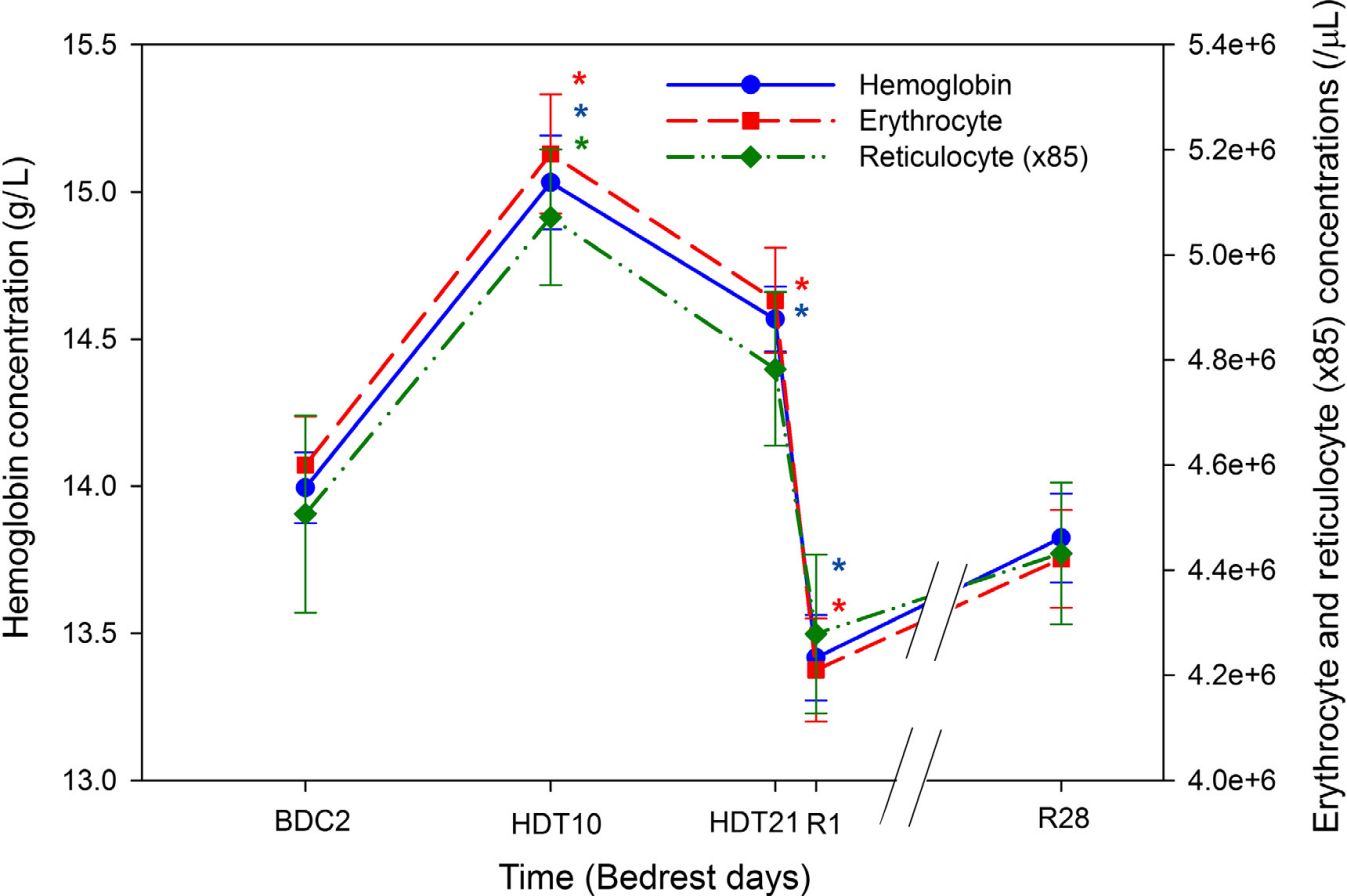
Hemoglobin, erythrocyte, and reticulocyte concentrations before, during, and after bed rest. Subjects remained hemoconcentrated during the 21 days of bed rest. A loss of erythrocytes is uncovered at reambulation day 1, with lower concentrations of hemoglobin and erythrocytes compared to baseline. The reticulocyte concentration was not significantly lower at R1 than at baseline. *P < 0.05 compared to baseline data collection (BDC). Reticulocyte concentrations were multiplied by 85 to be represented on the same Y2 axis as erythrocyte concentrations. Since the repeated measures ANOVA showed no effect of nutrition intervention (diet/control) or campaign (1 and 2) but a strong effect of duration of bed rest, data from all subjects are presented according to duration of bed rest. HDT: head down tilt; R: recovery.

Mean hemoglobin concentration (Hb) was increased at HDT10 (15.0 ± 0.2 g/L) and HDT21 (14.6 ± 0.1 g/L) compared to BDC2 (14.0 ± .01 g/L; both *P*s < 0.05) (Fig. 2). Hb at R1 was lower than BDC21 by 1.2 g/L (13.4 ± 0.1 g/L) and was lower than at BDC2 (*P* < 0.05). Erythrocyte concentration and mature erythrocyte concentration were higher at HDT10 (5.18 ± 0.06E6/*μ*L and 5.12 ± 0.06E6/*μ*L, respectively) and HDT21 (5.02 ± 0.06E6/*μ*L and 4.97 ± 0.06E6/*μ*L, respectively) compared to BDC2 (all *P*s < 0.05; Fig. 2). Erythrocyte and mature erythrocyte concentrations were lower at R1 (4.62 ± 0.06E6/*μ*L and 4.57 ± 0.06E6/*μ*L, respectively) compared to BDC2 (both *P*s < 0.05). Reticulocyte concentration was not decreased at R1 (5.03 ± 0.18E4/*μ*L) compared to BDC2 (5.30 ± 0.22E4/*μ*L; *P* < 0.05). At R28, Hb and erythrocyte concentrations had returned to BDC2 levels.

### Total hemoglobin mass and blood volumes

tHb decreased from 838 ± 21 g at BDC5 to 743 ± 19 g at HDT21 (*P* < 0.05). This 95 g decrease over 25 days corresponded to an average of 3.8 g/day. Blood volume decreased from 6462 ± 162 mL at BDC5 to 5621 ± 134 mL at HDT21 (*P* < 0.05). Plasma volume decreased from 4004 ± 111 mL at BDC5 to 3470 ± 82 mL at HDT21 (*P* < 0.05). Erythrocyte volume was lower at HDT21 (2157 ± 58 mL) compared to BDC5 (2459 ± 62 mL) (*P* < 0.05). At HDT21, the erythrocyte volume loss (302 ± 85 mL) had not yet matched the plasma volume loss (534 ± 138 mL), and the subjects were still hemoconcentrated.

### Numbers of red blood cell per subject

The number of erythrocytes and mature erythrocytes per subject was lower at HDT21 (2.56 ± 0.07E13 erythrocytes and 2.53 ± 0.07E13 mature erythrocytes) compared to baseline (2.90 ± 0.08E13 erythrocytes and 2.87 ± 0.08E13 mature erythrocytes) (both *P*s < 0.05). The number of reticulocytes at HDT21 (2.87 ± 0.1E11 reticulocytes) was also significantly lower than at baseline (3.17 ± 0.2E11 reticulocytes, *P* = 0.05). Assuming a reticulocyte life span of 1 day, a linear reduction in the reticulocyte production from BDC5 to HDT21, and a mean corpuscular hemoglobin of 29E‐12 g Hb/cell (Table 1), the decreased reticulocyte production contributed an estimated 0.4 g/day to tHb loss.

**Table 1:** Erythropoietic and hemolytic indicators before, during, and after bed rest

	BDC2 (1 SEM)	HDT10 (1 SEM)	HDT21 (1 SEM)	R1 (1 SEM)	R28 (1 SEM)
Hematocrit (%)	41.1 (0.4)	44.0 (0.5)^a^	42.5 (0.4)^a^	39.2 (0.5)^a^	40.5 (0.5)
Mean corpuscular volume (*μ*m^3^)	83.9 (0.4)^b^	84.9 (0.2)^b^	84.7 (0.5)^b^	84.7 (0.5)^a^,^b^	85.3 (0.5)^a^^,^ b
Mean corpuscular hemoglobin (pg)	28.9 (0.1)^b^	29.0 (0.2)^b^	29.0 (0.2)^b^	29.0 (0.2)^b^	29.2 (0.2)^a^
Mean corpuscular hemoglobin concentration (g/dL)	34.1 (0.1)^b^	34.2 (0.1)	34.3 (0.1)^a^	34.2 (0.1)^a^	34.2 (0.1)^a^^,^ b
Red cell distribution width (%)	12.9 (0.2)	13.3 (0.3)^a^	13.3 (0.2)^a^	13.2 (0.2)^a^	12.4 (0.1)^a^
Haptoglobin (g/L)	1.11 (0.14)	1.06 (0.13)	1.01 (0.10)	0.94 (0.09)	0.81 (0.07)^a^
Total bilirubin (mg/dL)	0.85 (0.08)	0.82 (0.08)	0.78 (0.08)	0.73 (0.08)	0.79 (0.08)
Direct bilirubin (mg/dL)	0.33 (0.03)	0.32 (0.02)	0.31 (0.02)	0.30 (0.02)	0.28 (0.02)
EPO (U/L)	10.8 (0.9)	10.0 (0.9)	11.8 (1.1)	14.5 (1.2)^a^^,^ b	14.6 (1.4)^a^^,^ b
Ferritin (*μ*g/L)	54.8 (7.1)	47.3 (6.4)	46.3 (5.8)	40.7 (5.3)^a^	18.6 (2.2)^a^
C‐reactive protein (mg/L)	0.22 (0.13)	0.09 (0.04)	0.19 (0.11)	0.39 (0.12)	0.10 (0.03)

a *P* < 0.05 compared to baseline data collection.

b *P* < 0.05 between campaign 1 and campaign 2.

### Hemolytic indicators

**Figure 3:**
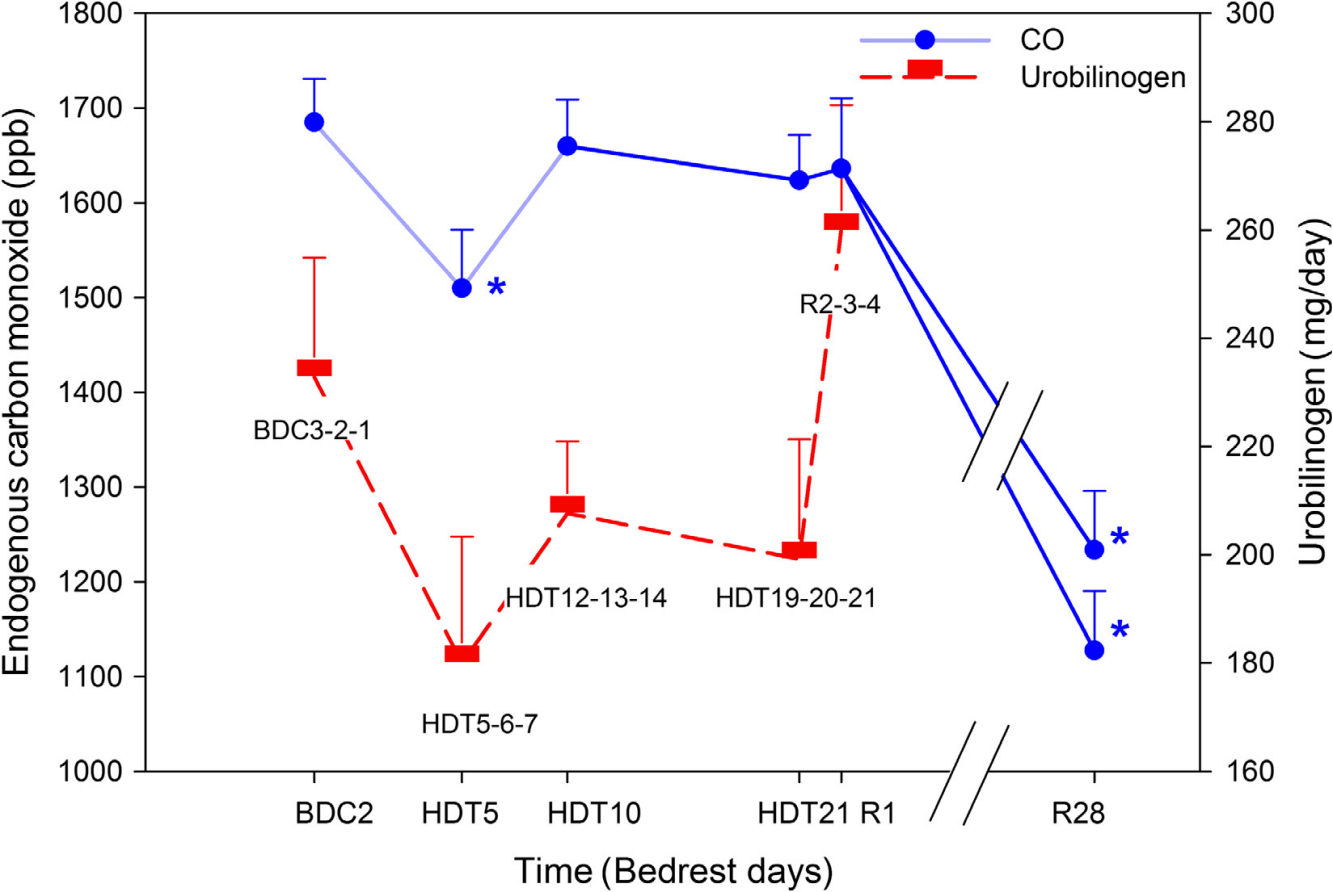
Endogenous [CO] and urobilinogen before, during, and after bed rest. Two direct measures of hemolysis, endogenous [CO] and urobilinogen, showed similar patterns. CO and urobilinogen constitute 1:1 end‐products from the heme oxidase catalysis of heme. No significant increase in [CO] or urobilinogen was measured during the hemoconcentrated state of bed rest compared to baseline. There was a significant decline in hemolysis at R28/R28repeat. There was also a significant decline in endogenous [CO] at HDT5, measured only in campaign 2 with *n* = 9. *P < 0.05 compared to baseline data collection (BDC). Stools were collected over 3 consecutive days, and average daily amount of urobilinogen is represented. ppb: parts per billion; HDT: head down tilt; R: recovery.

Mean endogenous [CO] showed no increase at HDT10 (1660 ± 49 ppb), HDT21 (1624 ± 48 ppb), or R1 (1636 ± 74 ppb) compared to baseline (1685 ± 46 ppb) (*P* > 0.05; Fig. 3). Significant decreases in endogenous [CO] were measured at times R28 and R28repeat (R28: 1127 ± 63 ppb and R28repeat: 1233 ± 62 ppb; both *P*s < 0.05 compared to baseline; Fig. 3). Endogenous [CO] at the additional time of HDT5 of campaign 2 (1510 ± 62 ppb; *n* = 9) was lower than at baseline (*P* < 0.05).

Mean fecal and urinary daily urobilinogen were unchanged during bed rest at HDT4‐5‐6 (180 ± 23 mg/day), HDT11‐12‐13 (208 ± 13 mg/day), HDT18‐19‐20 (199 ± 22 mg/day), and after bed rest at R1‐2‐3 (260 ± 23 mg/day) compared to baseline BDC3‐2‐1 (233 ± 23 mg/day; all *P*s > 0.05; Fig. 3). Urinary urobilinogen contributed negligibly (0.06 ± .02 mg/day) to the total urobilinogen.

Haptoglobin was unchanged during bed rest and lower at R28 compared to baseline (*P* < 0.05; Table 1). Total bilirubin and direct bilirubin were unchanged during bed rest. The decrease in total and direct bilirubin at R1 and R28 did not reach statistical significance (Table 1). Ferritin was lower at R1 and R28 compared to baseline (both *P*s < 0.05; Table 1).

### Other hematological indicators

EPO was increased at R1 and R28 compared to baseline (both *P*s < 0.05; Table 1). C‐reactive protein was unchanged during and after bed rest (Table 1).

## Discussion

Twenty‐one days of HDT bed rest caused hemoconcentration with loss of erythrocytes. We investigated the potential contribution of enhanced hemolysis in response to hemoconcentration. The two metabolites of the heme oxidase enzyme degradation of hemoglobin, endogenous [CO] and urobilinogen, followed a similar profile before, during, and after bed rest and showed no increased hemolysis. After bed rest, decreased hemolysis constituted a novel and interesting mechanism associated with the recovery from erythrocyte loss.

In healthy adults, ~2.5 × 10E11 new erythrocytes are produced and senescent erythrocytes are destroyed daily creating a steady state of heme degradation (Higgins and Mahadevan 2010). Expiratory CO has previously been used to measure the normal steady state of hemolysis as well as enhanced hemolysis in pathological conditions mainly in pediatric populations (Engstedt 1957; Sylvester et al. (2005); James et al. 2010; Caboot et al. 2012; Shih et al. 2014; Lal et al. 2015; Lozar‐Krivec et al. 2016). Tidmarsh et al. (2014) reviewed 13 studies that used end‐tidal CO as a marker of hemolysis and stressed for need for an accurate, sensitive, and reliable measurement device. The current study measured alveolar CO at the ppb precision, more sensitive than previous methods reporting 0.1 ppm precision, and reported no significant changes in CO elimination in the hemoconcentrated state of bed rest.

Increased hemolysis has previously been correlated with hyperbilirubinemia (Berlin 1975; Hampson 2007). In the current study, there was no increase in bilirubin levels during 21 days of hemoconcentration from bed rest. Enhanced hemolysis and increased bilirubin levels are not always synchronous: neonates with high end‐tidal CO later developed hyperbilirubinemia (Okuyama et al. 2001; Maisels and Kring 2006).

The final products of bilirubin degradation, urobilinogens, have a main mode of elimination through the intestinal tract, with less than 2% of urobilinogen excreted in the urine (Kotal and Fevery 1991). In the current study, we detected no increase in urobilinogen elimination during the hemoconcentration of bed rest. Haptoglobin binds with high affinity to free hemoglobin (Shih et al. 2014), and an increased ferritin concentration has been associated with significant hemolysis (Brabec et al. 1990); both haptoglobin and ferritin concentrations remained unchanged during bed rest in the current study.

The absence of an increase in multiple direct and indirect indicators of hemolysis at 5, 10, and 21 days of HDT bed rest confirmed our first hypothesis that bed rest caused hemoconcentration without a significant increase in hemolysis.

**Figure 4:**
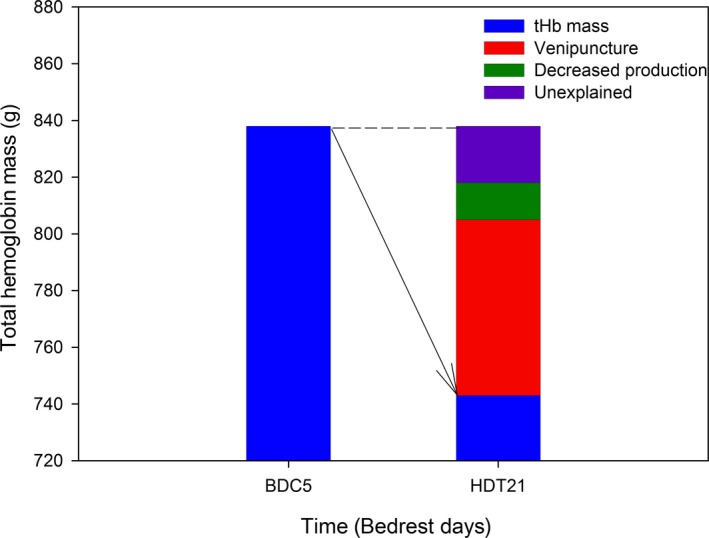
tHb loss during bed rest. tHb declined between baseline data collection day 5 (BDC5) and the end of 21 days of head‐down‐tilt bed rest (HDT21). The contributors to this decline in tHb included venipuncture and calculated decrease in erythrocyte production. Hemolysis of the unexplained portion of the tHb loss would have contributed a 185 ppb increase in steady‐state endogenous [CO] during bed rest.

What, then, accounted for the loss of tHb with 21 days of HDT bed rest? The tHb loss of ~3.8 g/day originated from various sources (Fig. 4). First, the blood draws needed for the various experiments contributed a majority of the tHb loss (~2.5 g/day). In addition, decrease in production was estimated at ~0.4 g/day. These two sources of tHb loss removed erythrocytes as a substrate for a putative hemolytic process. The loss of 0.9 g/day of hemoglobin remained unexplained. If 0.9 g/day of tHb were hemolysed, what would have been the predicted increase in steady‐state [CO] endogenous elimination? From the biochemical pathway of Hb degradation into CO, an increase of 185 ppb[CO] would be expected; well within the sensitivity of the current detection methods. The gas chromatograph with reduction gas detector measured ambient air [CO] throughout the 205 days of the two campaigns, and it was stable within 19 ppb (campaign 1: 315 ± 12 ppb; campaign 2: 334 ± 10 ppb). Therefore, the measures of [CO] in this study appeared reproducible over time and sensitive to detect changes in [CO] had enhanced hemolysis occurred. Other possible sources of erythrocyte loss include bleeding during muscle biopsies and at venipuncture sites.

Was the decline in tHb due to a loss of mature or immature erythrocytes? The mature erythrocyte concentration was decreased at R1, and the number of mature erythrocytes was decreased at HDT21 (*P* < 0.0001). The immature erythrocyte (reticulocyte) concentration was not significantly lower at R1, and the number of reticulocytes was decreased at HDT21 (*P* = 0.05). These findings support the predominant loss of mature erythrocytes. The exact mechanisms for erythrocyte maturation and the trigger to clearance when approaching a life span of 120 days remain largely unknown (Higgins and Mahadevan 2010). The predominant loss of mature erythrocytes may be related to their sensitivity to oxidative stress (Ghashghaeinia et al. 2012). Aging erythrocytes are removed from the circulation by spleen and liver macrophages (De Back et al. 2014). Two studies reported declining expression of CD47 and CD147 by erythrocytes with increased erythrocyte age, and this differentiated mature from young erythrocytes (Coste et al. 2001; Khandelwal et al. 2007). Lower CD147 expression may trap erythrocytes in the spleen (Coste et al. 2001), whereas lower CD47 expression was linked to phagocytosis (Khandelwal et al. 2007).

### Reambulation after bed rest

The first day of reambulation after 21 days of HDT bed rest was characterized by a lower hemoglobin concentration. A change in Ht from 42.2% at HDT21 to 41.1% at R1 indicated an approximate gain of 151 ml of blood volume in less than 2 days (Dill and Costill 1974). At R1 and R28, the subjects had enhanced EPO levels, indicative of increased stimulation of erythrocyte production. At R28, the subjects had recovered normal reticulocyte and Hb concentrations. Interestingly, the decline of over 500 ppb in endogenous [CO] at R28 supported a decreased rate of hemolysis at reambulation and is a novel finding. These data confirmed our second hypothesis that erythrocyte loss regenerates 28 days after bed rest through enhanced stimulation but introduces a coexisting mechanism of decreased hemolysis. Decreased hemolysis at reambulation was supported by higher haptoglobin, lower ferritin, and nonstatistically significant decreases in total and direct bilirubin levels at R1 and R21. The predominant loss of mature erythrocytes during bed rest may have resulted in a younger erythrocyte population, with fewer erythrocytes entering senescence partly explaining the decreased hemolysis rate at R28. This possibility is supported by the higher mean corpuscular volume, mean corpuscular hemoglobin, and mean corpuscular hemoglobin concentration attributed to a younger erythrocyte population at R1 and R28 (Table 1).

Decreased hemolysis levels have been reported experimentally with various antioxidants in situations of oxidative stress, zinc deficiency, hypercholesterolemia, or medication intake (Ghoti et al. 2011; Kotsuruba et al. 2015; Liao et al. 2015). Rizzo et al. (2012) documented heightened antioxidative response of mice erythrocytes to microgravity, of which bed rest is a model.

The current study proposes the following sequence of erythrocyte adaptations to prolonged bed rest:

- During bed rest: loss of plasma volume → hemoconcentration → decline in erythrocyte numbers (predominantly mature erythrocytes) → no measurable increase in the rate of hemolysis → maintained EPO levels.
- After bed rest: gain in plasma volume → hemodilution → EPO stimulation to restore erythrocyte numbers and concentrations → decreased hemolysis.

Physicians in clinical practice should pay attention to the decreased tHb from prolonged bed rest. At patient reambulation, hemodilution may decrease the hemoglobin and erythrocyte concentrations. In the community, geriatric surveys have reported a high prevalence of unexplained anemia in populations with limited mobility or who are bedridden (Guralnik et al. 2004). Erythrocyte loss from prolonged bed rest may partly explain the high prevalence of unexplained anemia in people with decreased mobility (Guralnik et al. 2004).

The erythropoietic adaptations to bed rest bear similarities with and differences from other hemoconcentrated conditions. Rapid hemolysis of 10–15% of tHb within 10 days at the expense of newly formed erythrocytes has been reported in space, descending from altitude, in kidney failure and upon withdrawal from EPO administration (Alfrey et al. 1996, 1997; Rice et al. 1999, 2001; Chang et al. 2009; Pottgiesser et al. 2012). More recently, other groups have reported a progressive decrease in hemoglobin mass for weeks, similar to our findings, in microgravity, after EPO cessation and returning from high altitude (Pottgiesser et al. 2012; Durussel et al. 2013; Gore et al. 2013; Wachsmuth et al. 2013; Risso et al. 2014; Ryan et al. 2014). A meta‐analysis of 17 studies on descent from altitude concluded that “evidence for a rapid decrease in Hb mass is not present” (Gore et al. 2013). Reanalysis of datasets from early space flights revealed a similar removal rate of erythrocytes of all ages (Risso et al. 2014).

Practically, extreme environments such as space and high altitude pose experimental challenges, and most reports indirectly measured hemolysis (Franco 2009). The current study benefitted from direct measures of hemolysis in healthy men in laboratory conditions. Ryan et al. (2016) completed a 4‐day HDT bed rest study on seven subjects and reported a similar sequence of changes for most measures including decreased plasma volume, hemoconcentration during bed rest, and decreased tHb after bed rest. Interestingly, they also reported decreased hemolysis at 4 days of bed rest.

### Limitations

Erythrocytes removed through venipuncture and reticulocytes not produced are unavailable to hemolysis and were accounted for in our analysis. Despite this, the subjects remained hemoconcentrated throughout bed rest, supporting the validity of HDT bed rest to study hemoconcentration. Hemoconcentration during bed rest and hemodilution after bed rest can potentially affect hemolytic indicators. This was not the case since blood volume contraction would increase venous blood PCO and endogenous [CO] and overestimate hemolysis. In addition, no increases in bilirubin, EPO, or ferritin were measured during hemoconcentration. tHb and plasma volume calculations are based on physiological assumptions (Schmidt and Prommer 2005) that may be affected by HDT bed rest. First, degradation of nonhemoglobin–heme‐containing proteins was set at 15% of the CO production (Franco 2009; Coburn 2012). Lower tHb during bed rest may increase the proportion of CO originating from nonheme molecules, overestimating the hemolysis rate. Second, muscle atrophy and protein loss during bed rest may alter the hematocrit in muscle capillary flow (Poole et al. 2013) and CO kinetics between hemoglobin and myoglobin (Lee et al. 2013). However, both decreases and increases in myoglobin content have been described with muscle atrophy (Lee et al. 2013; Turner et al. 2014). Third, bed rest may alter alveolar ventilation (Saltin 1968; Sandler and Verniko 1986) and hypoventilation would increase venous pCO and endogenous [CO] and overestimate hemolysis. Fourth, diffusion of CO decreased 20% with 120 days of bed rest (Montmerle et al. 2002). Fifth, bed rest may alter the Fahraeus correction factor of 0.91 (Schmidt and Prommer 2005). Finally, it has been suggested that systemic inflammation may increase CO elimination, however no correlation existed between sepsis and HbCO (McArdle et al. 2016) and inflammatory marker C‐reactive protein was stable in this study. Limitations of the CO‐rebreathing method to measure tHb mass was mitigated by using venous blood samples collected from the same anatomical site, with patients in the same posture and analyzed by the same staff using the same blood gas machines. Otto et al. (2017) reported a typical error of 1.93%, comparable to other studies (Sandler and Verniko 1986; Weber et al. 2007). Given the change in tHb mass measured in the current study (11%), the CO‐rebreathing method limitations do not weaken the conclusions.

## Conclusions

This study provides the first direct evidence for the absence of accrued hemolysis during the hemoconcentrated state of 21 days of HDT bed rest. Interestingly, this study identified reduced hemolysis rates in the reambulation phase after bed rest that accompanied enhanced EPO levels to restore erythrocyte concentration. The findings may apply to other hemoconcentrated states such as astronauts, bedridden patients, elite athletes training in altitude, and EPO administration. Our findings support further research into mechanisms for resolution of hemoconcentrated states and for modulation of hemolysis in the recovery from various types of anemia.

## Conflict of Interest

The authors have no competing interests to disclose.

## Data Accessibility

The minimal dataset underlying the figures provided in the paper that is necessary to support the central findings of the study, and to interpret, analyze, or reproduce the methods and findings will be made available upon request.
